# Potential of near-infrared fluorescence image-guided debridement in trauma surgery

**DOI:** 10.1080/23320885.2018.1481410

**Published:** 2018-06-28

**Authors:** Tim Pruimboom, Rutger M. Schols, Shan S. Qiu, René R.W.J. van der Hulst

**Affiliations:** Department of Plastic, Reconstructive and Hand Surgery, Maastricht University Medical Center, Maastricht, The Netherlands

## Abstract

This case report presents the use of near-infrared fluorescence (NIRF) imaging using indocyanine green (ICG) and its potential for the evaluation of soft tissue viability in a traumatic case. Standard implementation of this novel imaging modality might decrease the number of surgical debridement procedures in complex traumatic wounds.

## Graphical abstract

**Figure:**
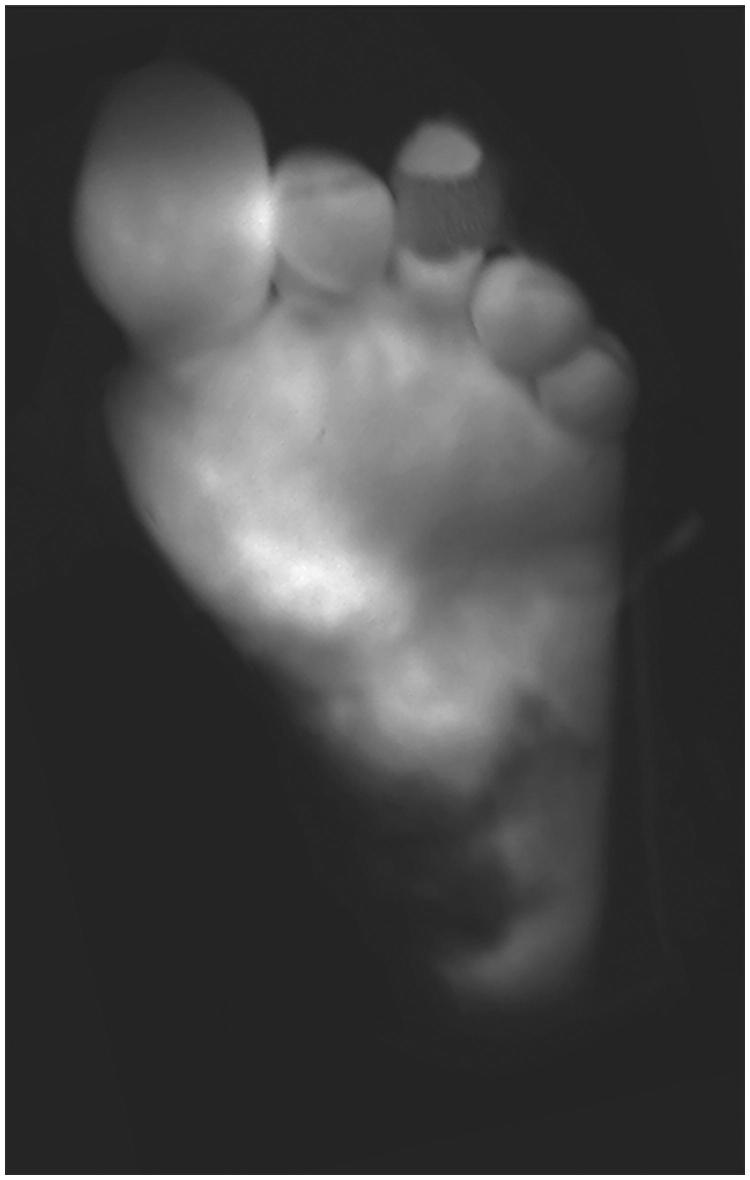

## Introduction

Adequate and early evaluation of tissue viability is of great importance in the treatment of open fractures of an extremity with comprehensive soft-tissue defects [1]. Early, thorough debridement of all non-viable tissue is essential in treating these fractures since inadequate initial debridement may be accompanied by an increased risk of infection and nonunion [2]. Final closure strategy should be postponed until the wound is ‘stabilized’, defined by the presence of a viable wound bed and the absence of necrotic tissue. Assessment of the viability of soft tissues and bone is generally based on the discretion of the surgeon’s subjective judgement [3].

Near-infrared fluorescence (NIRF) imaging can provide a more objective assessment of (soft) tissue viability. After intravenous administration of a contrast agent with fluorescent characteristics in the near-infrared (NIR) light spectrum (e.g. indocyanine green (ICG)) the surgeon can capture real-time enhanced tissue detection and characterization using a dedicated camera system. This innovative imaging method allows for in-depth (maximum penetration depth of up to 1.5 centimeter) visualization of target-tissues including a wide variety of anatomical structures [4]. NIRF is widely used by plastic surgeons to assess tissue perfusion in free flap surgeries and to study the lymphatic system [5–7].

With the current report the potential added clinical value of NIRF imaging in combination with ICG is illustrated for enhanced debridement of traumatic wounds in the lower extremity.

## Case report

**Figure 1.**
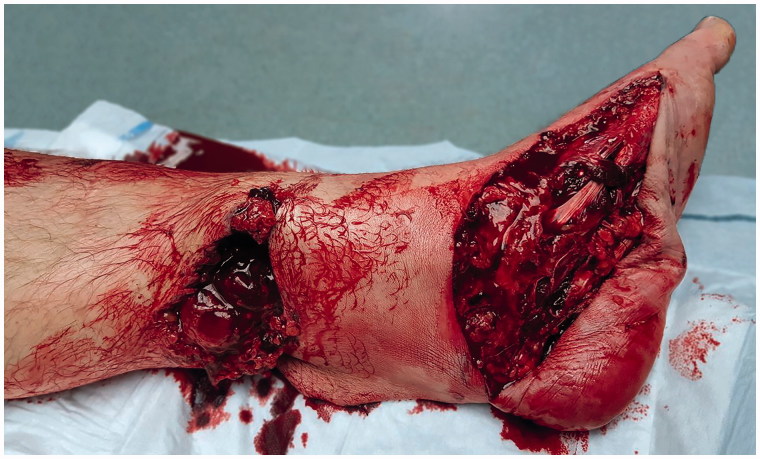
Preoperative aspect of the wound in the left lower leg (Soft tissue defect on the medial arch of the foot, foot sole and medial side of the lower leg).

**Figure 2.**
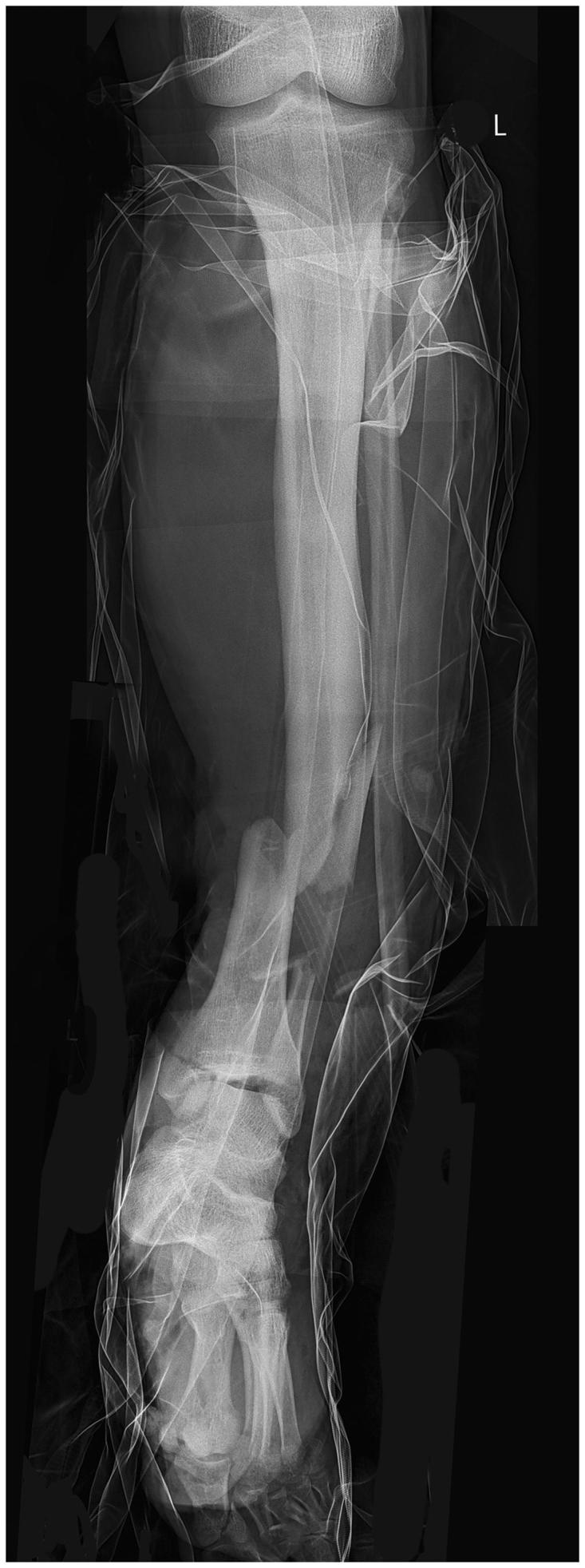
X-ray photo of the bone fractures.

A healthy 40-year-old man presented at the emergency room after a high energetic accident with multiple fractures and comprehensive soft tissue defects on his left lower leg and foot. See Figure 1. After clinical examination in combination with X-ray images and CT-scan of his left lower leg the following fractures were identified: a Gustilo grade IIIB fracture, a medial malleolus fracture, a Tillaux-Chaput fracture, a nutcracker fracture of the cuboid, an avulsion fracture of the navicular bone, fractures of the third, fourth and fifth metatarsal head, a mid-shaft fracture of the third proximal phalanx and base fractures of the first and second proximal phalanx. See Figure 2. In Supplemental Online Figure 1 the CT-scan of the left lower leg and foot is displayed. Active extension and flexion of the toes were preserved, as well as sensibility in the toes and forefoot. Disturbed sensibility to touch and pain was identified at the foot sole. No signs of a compartment syndrome were found.

**Figure 3.**
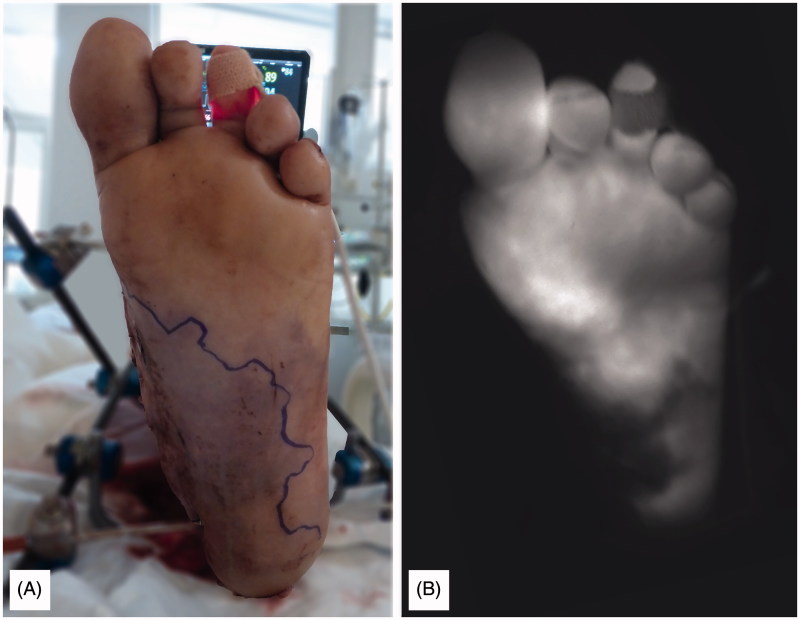
Marked edges of non-viable tissue and clinical aspect of skin combined with NIRF imaging. (A: conventional “white-light” image; B: NIRF imaging).

Within the first thirteen hours after admission placement of an external fixator and debridement of the proximal medial wound was conducted. The soft tissue defects were temporarily covered with Epigard^TM^ (Biovision, Ilmenau, Germany) and absorbent dressings. Within the first 24 hours postoperatively NIRF imaging was performed. See Figure 3 and Supplemental Online Figure 2.

The imaging head of the Fluobeam^TM^ (Fluoptics, Grenoble, France) was positioned approximately 8 inches above the skin. Real-time images were obtained during 120 seconds, starting 10 seconds after intravenous injection of 2 mL (5 mg) of ICG. The fluorescent region (white area) indicates the area of vital tissue. Regions of no fluorescence (black area) and less fluorescence (surrounding grey area) were marked to indicate the area of non-viable tissue.

The patient underwent a second debridement approximately 2.5 days after initial admission. The remaining defects were covered with Epigard^TM^ and absorbent dressings. Postoperative non-viable skin was marked again guided by NIRF imaging. Remarkably, a slight increase of the fluorescent area was visualized at the lateral malleolus. A final debridement was performed 6 days after hospital admission in the same session as definitive soft tissue coverage. The remaining avital soft tissue was removed under real-time intraoperative NIRF image-guidance. Intraoperative imaging showed no difference in the extension of the fluorescent area compared to the previous NIRF imaging. A free anterolateral thigh flap was harvested to cover the defect on the medial malleolus and foot sole. Additional split skin graft was placed to cover the superficial defect at the lateral malleolus. No complications were recorded and the patient was discharged after five weeks. Nine weeks postoperatively the patient and flap were doing well. Clinical rehabilitation care is completed and he mobilizes with crutches. See Supplemental Online Figures 3, 4 and 5 for illustration of the clinical course of the lower leg and foot.

## Discussion

This case illustrates the potential of NIRF imaging using ICG to assist accurate debridement of complex traumatic wounds of the lower extremity. NIRF imaging might decrease the number of surgical debridement procedures before final reconstruction can be performed. In this case, the patient could have benefited from less number of surgical debridement procedures if they would have been performed under the guidance of NIRF imaging, allowing surgeons to be more radical and precise. This case supports the conclusion of Koshimune et al. [1] who applied the NIRF imaging technique in the reconstruction of Gustilo grade IIIB open lower-limb fractures to rule out the presence of necrotic tissue and to determine the margins for debridement.

Furthermore, NIRF angiography might aid in early detection of compromised vascularization before clinical changes become evident. The described case shows a good correlation between clinical course and NIRF imaging. The images did not substantially change from the first day to one week after the accident and therefore proved to be more reliable compared to the empirical evaluation. However, subtle differences in fluorescence demarcation could be seen in the area ventral to the lateral malleolus between the first and second assessment. This is probably due to slight differences in timing between the intravenous ICG administration and the acquisition of NIRF imaging. Therefore, a standard protocol in terms of timing for the performance of NIRF imaging should be determined with a larger number of patients.

One of the important limitations of NIRF imaging is the difficulty to describe the precise cut-off point in the border zone (grey area) between viable and non-viable tissue. Normally the ‘grey area’ surrounds the ‘black area’ for a maximum of 1–1.5 cm in width. In this case both areas are marked. In case of doubt, clinical correlation is necessary by making superficial cuts in the skin to check perfusion.

NIRF imaging devices are relatively expensive (approximately $40.000) [8], but the costs can easily be compensated by reduction of debridement procedures and related healthcare costs (e.g. operation room fee and costs for additional days in hospital). Moreover, in a fast growing amount of hospitals a NIRF imaging device is already available, as it has been purchased for other procedures (e.g. lymphatic surgery, free flap surgery, oncological surgery). In the aforementioned situation, remaining costs are only $20 (i.e. maximum costs for 5 mg ICG for infusion) per assessment, making it more cost-efficient by reducing debridement procedures.

In conclusion, further clinical studies are warranted to assess the appropriate timing of performing NIRF imaging and to obtain generalized quantification methods of NIRF images, hence determining a precise cut-off point. Consequently, the possible advantages of NIRF imaging in terms of cost-effectiveness in trauma surgery need to be determined.

## Supplementary Material

Supplemental Online Figure 5

Supplemental Online Figure 4

Supplemental Online Figure 3

Supplemental Online Figure 2

Supplemental Online Figure 1
