# Supplementary figures and images for: Potential of near-infrared fluorescence image-guided debridement in trauma surgery
Source: Case Reports Plast Surg Hand Surg. 2018 Jun 28;5(1):41–4. doi: 10.1080/23320885.2018.1481410 (PMC6032015; doi:10.1080/23320885.2018.1481410)

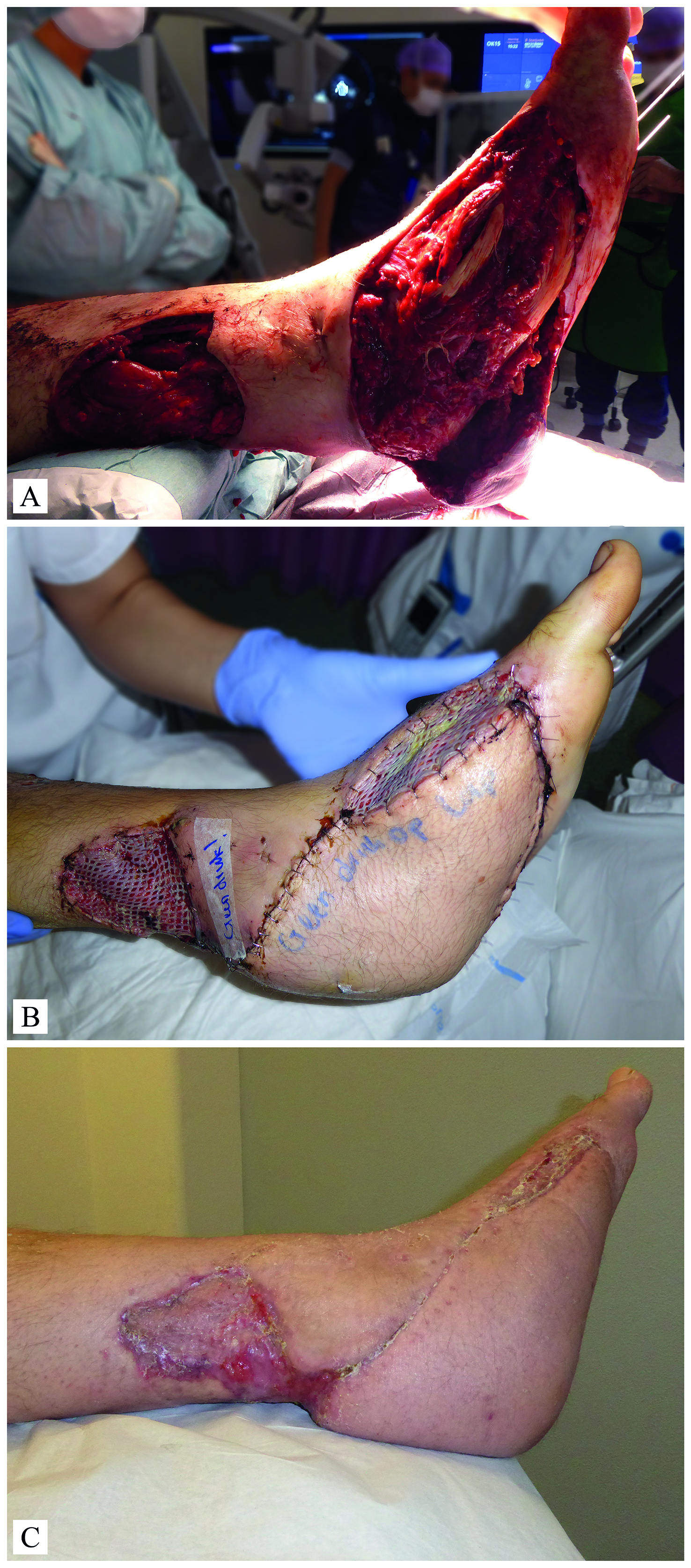

Supplement: Supplemental Online Figure 5 [file ICRP_A_1481410_SM7680.tif]

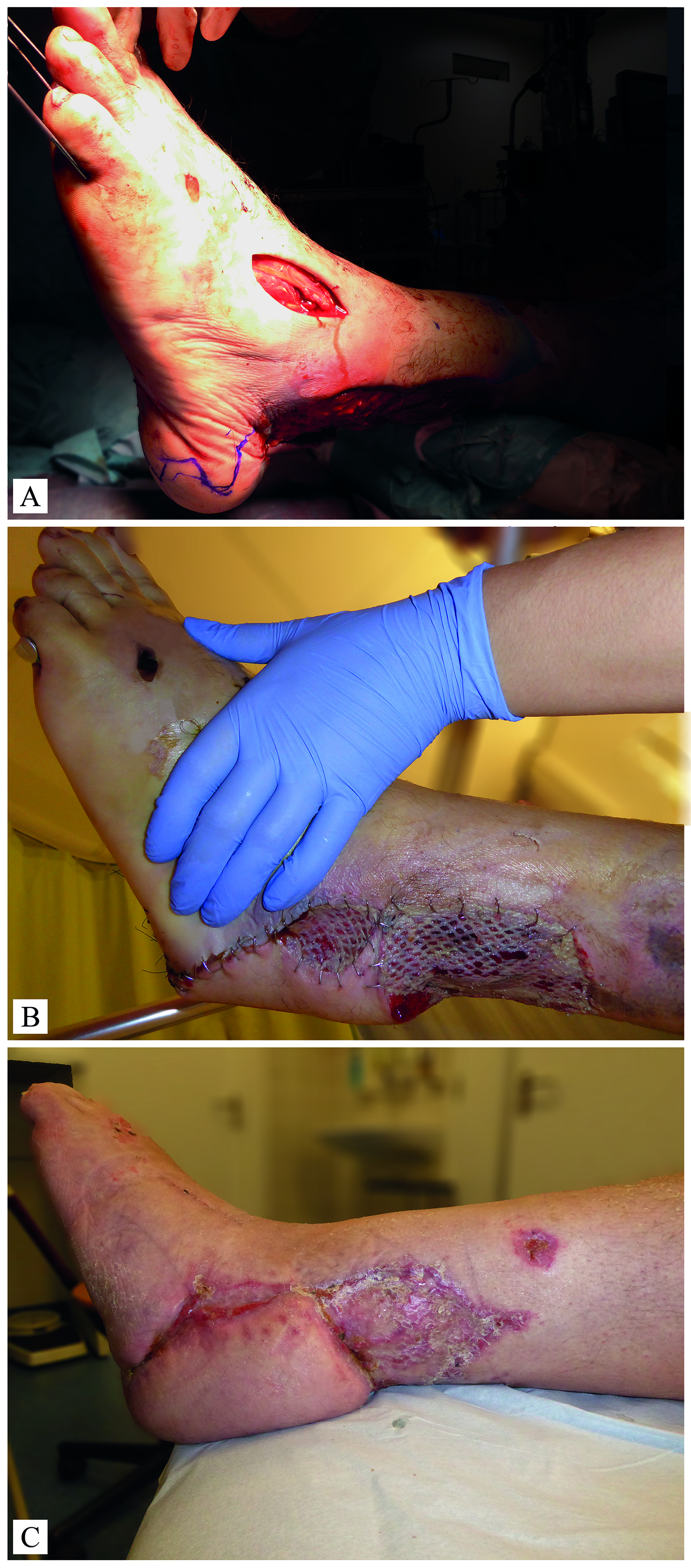

Supplement: Supplemental Online Figure 4 [file ICRP_A_1481410_SM7679.tif]

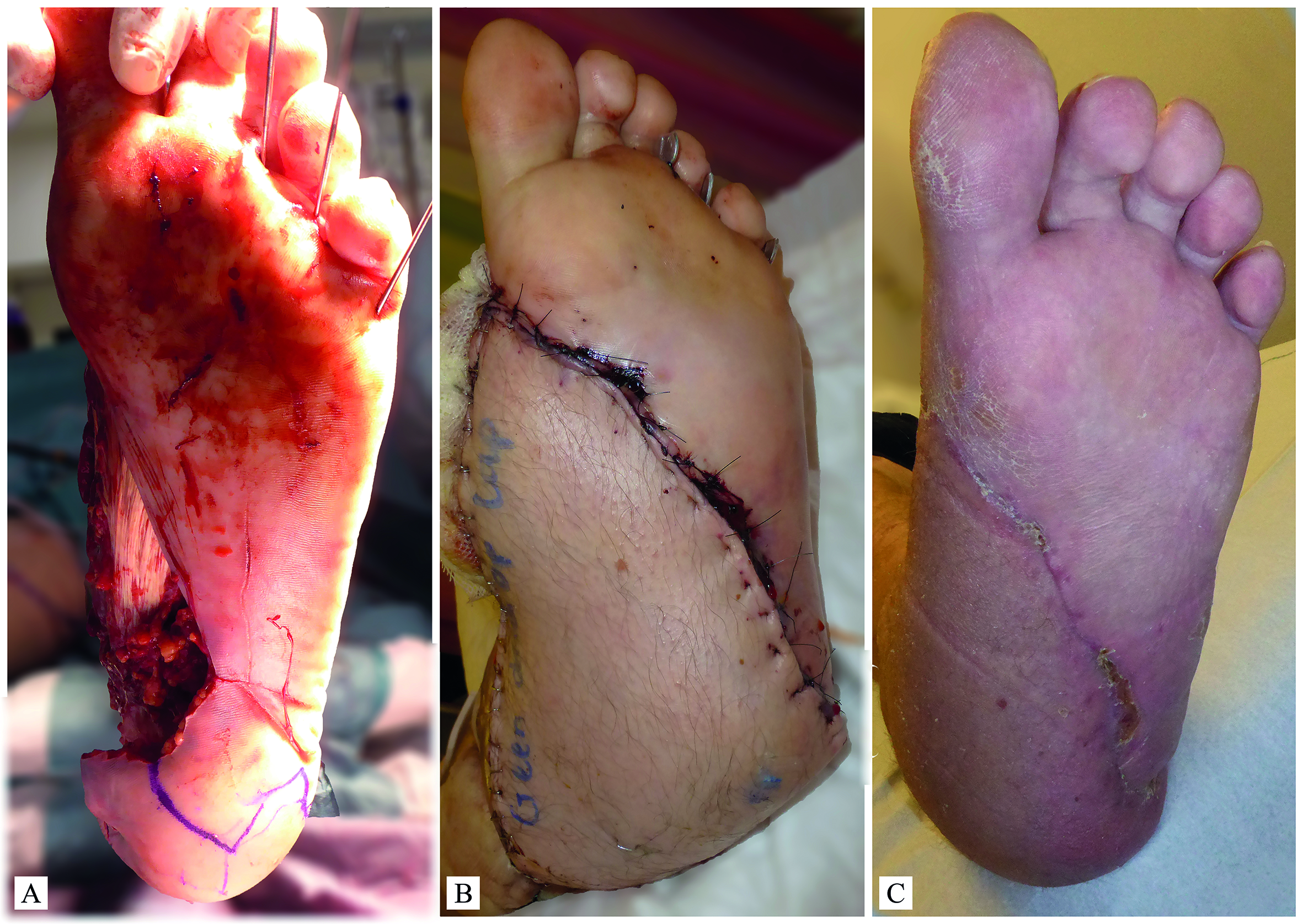

Supplement: Supplemental Online Figure 3 [file ICRP_A_1481410_SM7678.tif]

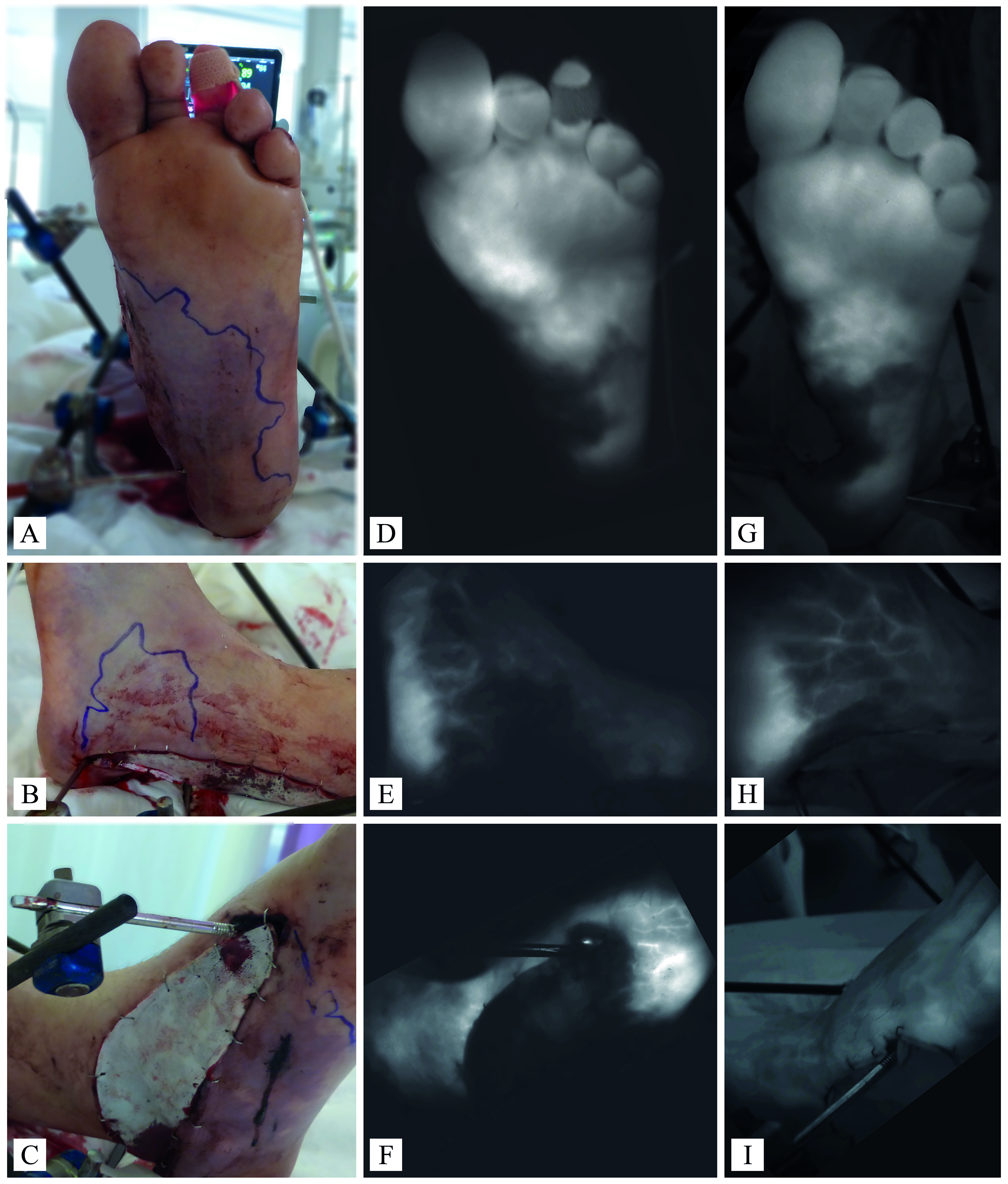

Supplement: Supplemental Online Figure 2 [file ICRP_A_1481410_SM7677.tif]

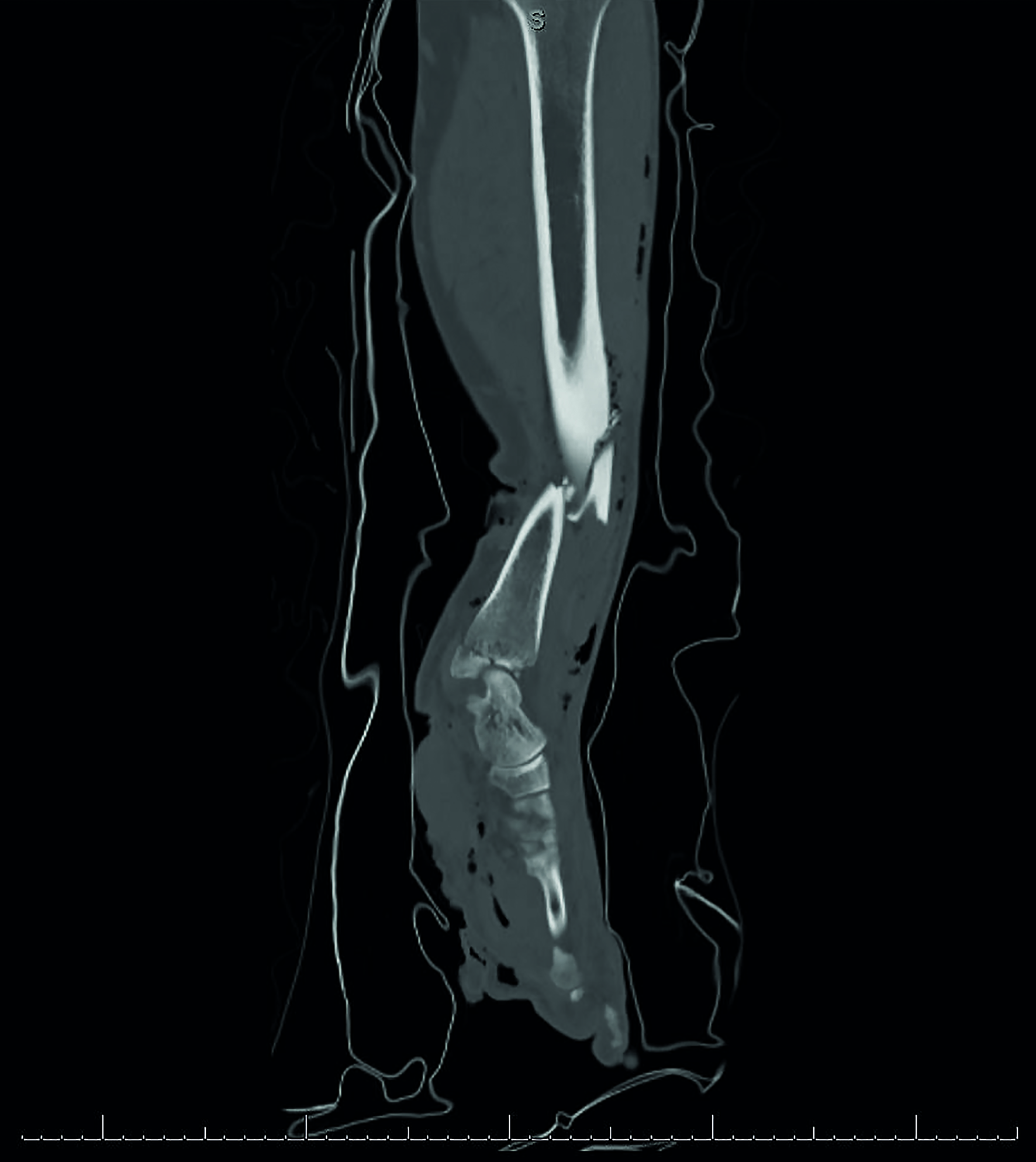

Supplement: Supplemental Online Figure 1 [file ICRP_A_1481410_SM7676.tif]
